# Macrophages participate in host protection and the disease pathology associated with *Leishmania braziliensis *infection

**DOI:** 10.1186/1471-2334-12-75

**Published:** 2012-03-29

**Authors:** Angela Giudice, Célia Vendrame, Caroline Bezerra, Lucas P Carvalho, Thaís Delavechia, Edgar M Carvalho, Olívia Bacellar

**Affiliations:** 1 Serviço de Imunologia, Complexo Hospitalar Universitário Professor Edgard Santos, Universidade Federal da Bahia, Salvador, Bahia, Brazil; 2 Instituto Nacional de Ciência e Tecnologia de Doenças Tropicais- INCT-DT(CNPq/MCT), Salvador, Bahia, Brazil; 3 Complexo Hospitalar Universitário Prof. Edgard Santos, Serviço de Imunologia, 5o andar, Rua João das Botas, s/n, Canela, 40110-160 Salvador, BA, Brazil

**Keywords:** *L. braziliensis*, Macrophages, Chemokines, Subclinical infection

## Abstract

**Background:**

*Leishmania *preferentially infects macrophages, which allow the parasite to multiply but can also kill the parasite. Although the T cell response in human leishmaniasis is well-characterized, little is known about the concomitant macrophage behavior. The aim of this study was to characterize the macrophage immune response after *Leishmania braziliensis *infection in cells derived from cutaneous leishmaniasis (CL) or mucosal leishmaniasis (ML) patients, subclinical individuals (SC) and healthy control subjects (HS).

**Methods:**

Peripheral blood mononuclear cell-derived macrophages from the different groups were exposed to *L. braziliensis in vitro *and were evaluated for susceptibility to *Leishmania *infection, ability to kill *Leishmania *and chemokine/cytokine production. Nitric Oxide (NO) and superoxide (O_2_^-^) levels in the supernatant of infected macrophage cultures were monitored.

**Results:**

After exposure to *L. braziliensis*, peripheral blood mononuclear cell-derived macrophages from SC individuals showed a lower infection rate and a smaller number of intracellular amastigotes compared to cells from CL and ML patients. Macrophages from CL and ML patients produced more chemokines and TNF-α than those from the SC group. Production of NO and O_2_^- ^were detected but did not vary significantly among the different groups.

**Conclusions:**

Our data indicate that macrophages play a pivotal role in controlling *L. braziliensis *infection and in leishmaniasis pathology by secreting pro-inflammatory chemokines/cytokines that activate and recruit T cells, overwhelming the inflammatory response.

## Background

*Leishmania Viannia braziliensis *is the primary cause of American Tegumentary Leishmaniasis (ATL) in Brazil. Cutaneous leishmaniasis (CL), the most common form of ATL, is characterized by the appearance of a single or a few ulcerated skin lesions. A fraction (3%) of CL patients develops mucosal disease, characterized by disfiguring lesions that usually involve the nasal or oropharyngeal mucosa [1,2]. CL and ML lesion biopsies show an intense inflammatory reaction, characterized by a predominance of lymphocytes and macrophages and few or no detectable parasites [1]. In areas of endemic *Leishmania *transmission, approximately 10% of individuals have a positive Montenegro skin test (MST) but lack disease pathology [3-6]. These individuals are classified as subclinical (SC) or asymptomatic infection cases. The immunological response in CL and ML has been well-characterized. The type-1 immune response predominates in CL and ML, with an exaggerated production of IFN-γ and TNF-α that fails to be modulated either by IL-10 or TGF-β or by neutralizing antibodies to IL-12 and IL-15 [7,8]. Lymphocytes from ML patients proliferate more than those from CL patients when stimulated with parasite antigens and produce higher levels of IFN-γ and TNF-α [7,9]. This exacerbated type-1 immune response is unable to control the disease and contributes to pathological tissue damage. In contrast, SC patients produce only low levels of IFN-γ and TNF-α [10].

Macrophages, the host cells for the intracellular parasite *Leishmania*, are crucial for the outcome of disease. However, very little is known about macrophage behavior in human *Leishmania *infection. In addition to presenting antigens to T cells, macrophages secret chemokines and cytokines involved in the inflammatory response, such as IL-1β and TNF-α in response to *Leishmania *infection. Lesions from CL patients show increased expression of CCL2, CXCL9 and CXCL10 [11]. CCL2 and CCL3 are known to increase the leishmanicidal ability of human macrophages to the same level induced by IFN-γ [12]. However, molecules such CXCL9, CXCL10 and TNF-α, which are highly expressed in the tissue from CL and ML patients, participate in the inflammatory reaction that may lead to the tissue damage observed in CL and ML [11,13].

When *Leishmania *parasites enter host macrophages, an oxidative burst occurs that is characterized by an increase in reactive oxygen species and reactive nitrogen intermediates, such as nitric oxide (NO). The mechanisms used by human macrophages to kill *Leishmania *are not yet well established. While NO production plays a role in killing *Leishmania *in mice, its role in humans is still questionable [14-16].

The aim of the present study was to characterize the immune response of macrophages from CL, ML, and SC patients and from healthy subjects (HS) after exposure to *Leishmania*. We evaluated the susceptibility of macrophages to infection by *L. braziliensis *and the ability of these cells to kill *Leishmania*. Additionally, we investigated the role of NO and O_2_^- ^in killing the *L. braziliensis *parasite. We also evaluated the production of chemokines (CCL2, CCL3, CXCL8 and CXCL9) and TNF-α.

## Methods

### Subjects

This study included patients with CL (n = 22), ML (n = 11) or SC (n = 18) and HS controls (n = 14). All patients were seen at Corte de Pedra Health Post, located in the southeast region of the State of Bahia, Brazil, which is well known for its high rate of *L. braziliensis *transmission. Some experiments did not include samples from every patient because some of the experiments in this study required a larger number of cells than could be obtained from the patients. The diagnostic criteria were a clinical picture characteristic of CL or ML, parasite isolation or a positive delayed-type hypersensitivity (DTH) response to leishmania antigen, and histological features of CL or ML. Household contacts of ATL patients without past or current history of leishmaniasis with a positive DTH test and/or IFN-γ production in cultures stimulated with SLA were considered as having subclinical infection. The physical examination of these individuals did not show a typical scar of CL. Immunological analyses were performed prior to therapy for all patients. Healthy subjects were composed of students at the Federal University of Bahia.

This study was approved by the Ethical Committee of the Federal University of Bahia. Informed consent was obtained from all enrolled subjects.

### Parasites

A *L. braziliensis *(MHOM/BR/2003/LTCP15344) isolate obtained from a skin lesion on a CL patient was initially cultivated in biphasic medium (NNN). Following isolation, the parasites were cryopreserved in frozen nitrogen. The parasites selected for this study had not been previously passaged in liquid culture medium. After selection, the parasites were expanded in complete Schneider's medium. The isolate was identified as *L. braziliensis *by multilocus enzyme electrophoresis [17].

### Soluble leishmania antigen (SLA)

The leishmania lysate (crude antigen) was prepared from a *L. braziliensis *strain (MHOM/BR/2001) as previously described [18].

### Cell separation and macrophage culture

Peripheral blood mononuclear cells (PBMCs) were isolated from heparinized blood using Ficoll Hypaque (LSM; Organon, Durham, NC). Monocytes were separated by adherence. After 6 days of culture in complete RPMI 1640 (10% heat inactivated human AB serum, 100 U penicillin/ml, 100 μg streptomycin/ml) (GIBCO BRL, Grand Island, NY, USA), the adherent cells displayed characteristics of monocytes-derived macrophages.

More than 99% of the cells in culture were identified as macrophages by microscopic observation. To assess purity of macrophages, adherent cells were removed by harvesting and analyzed by flow cytometry. Macrophages were gated according to their characteristic forward- and side-scatter. Purity was assessed by analysis using the following conjugated mouse anti-human mabs: CD14-PE-Cy5 (macrophages), CD3-PE (T cells), CD19- FITC (B cells), and were confirmed to be CD14^+^, CD3^-^, CD19^-^. The T cell influence on the system was considered to be absent because the adherent cell population consisted of less than 1% CD3^+ ^T cells (data not shown). We also evaluated the production of IFN-γ in supernatants of infected macrophages by ELISA and observed that the IFN-γ levels were very low or undetectable.

### Macrophage infection by *L. braziliensis *and evaluation of chemokines and TNF-α production

PBMC-derived macrophages from HS and from SC, CL and ML patients were infected with *L. braziliensis *stationary phase promastigotes at a 2:1 ratio. Uninfected macrophages and LPS-stimulated macrophages (100 ng/ml) were used as controls. Infected macrophages were incubated at 37°C in 5% CO_2 _for 2 h. After incubation, the remaining extracellular parasites were removed by gentle washing. Cells were stained with Giemsa stain after 2, 48 and 96 h of infection, and the infection levels were determined by counting the percentage of infected cells and the number of amastigotes per 100 macrophages. This analysis was performed by two independent observers who were blinded to the experimental conditions. After each timepoint, supernatants were harvested and stored at -70°C for analysis of chemokines, TNF-α NO and O_2_^- ^production.

### Determination of Nitric Oxide and superoxide production

Nitrite (NO_2_^-^) accumulation in the cell culture supernatants was used as an indicator of NO production and was determined by a standard Griess reaction [19]. Superoxide production (O_2_^-^) was determined by adding hydroxylamine (0.5 mM) to cultures of infected macrophages [20,21]. Hydroxylamine converts superoxide into nitrite, which can be quantified by performing the Griess reaction, as described above. Background levels of nitrite generated by the release of NO were determined in parallel, without the addition of hydroxylamine.

### Determination of chemokines and TNF-α production

The cell culture supernatants from HS, SC, CL and ML patients were assayed for CCL2, CCL3, CXCL8, CXCL9 and TNF-α after 2, 48 and 96 h of infection by ELISA (BD Pharmingen and R&D Systems, Minneapolis, MN). Purified cytokines were used to derive a standard curve. The sensitivity of the cytokine assays was 15.6 pg/ml (CXCL8), 15.6 pg/ml (CXCL9), 7.8 pg/ml (CCL2), 7.8 pg/ml (CCL3) and 15.6 pg/ml (TNF-α).

### Statistical analysis

Kruskal-Wallis with Dunn's post-test was used to analyze the results from the macrophage infection experiments and to compare the levels of chemokines TNF-α, NO and O_2_^- ^produced by macrophages. The cut-off for statistical significance was set at *p *< 0.05. Statistical analysis was performed using Grand Prism 3.0 (GraphPad software, San Diego, CA, USA)

## Results

### Susceptibility of human macrophages to *L. braziliensis *infection

**Figure 1:**
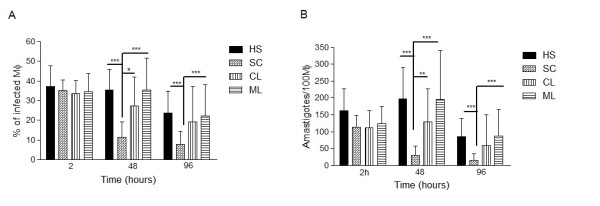
**Phagocytosis and killing of *L. braziliensis *by macrophages from different forms of *L. braziliensis *infection**. Human monocyte derived-macrophages from HS (n = 12), SC (n = 12), CL (n = 10) and ML (n = 11) were infected with *L. braziliensis *in the stationary phase of growth at a 2:1 ratio. The percentage of infected macrophages **(A) **and the parasite burden **(B) **were evaluated at 2, 48 and 96 h after infection. Data represent the mean ± SD. Statistical analysis was performed using the Kruskal-Wallis test. (* *p *< 0.05, ** *p *< 0.01, *** *p *< 0.001).

To determine the susceptibility of macrophages to *L. braziliensis*, as well as their ability to kill this parasite, adherent cells from the 4 different groups of subjects (HS, SC, CL and ML) were infected with *L. braziliensis *stationary phase promastigotes. The percentage of infected cells and the number of intracellular amastigotes/100 macrophages were similar in the groups at 2 h after infection (Figures 1A and 1B). After 48 h, SC macrophages had a lower proportion of infected cells and a lower number of amastigotes per 100 cells compared to HS, CL and ML macrophages (SC × HS, *p *< 0.001; SC × CL, *p *< 0.01; SC × ML, *p *< 0.001). After 96 h of infection, the percentage of infected macrophages and number of amastigotes per 100 cells declined in all four groups. However, both values were significantly lower in the SC patients compared to HS and ML patients *p* <0.001 (Figures 1A and 1B).

### *L. braziliensis *killing is not associated with NO and superoxide production

**Figure 2:**
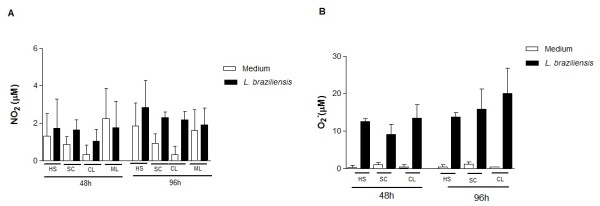
**Nitric Oxide (NO) and superoxide production by macrophages after infection with *L. braziliensis***. Six-day monocyte derived-macrophages from HS (n = 6), SC (n = 6), CL (n = 6) and ML (n = 05) groups were infected or left uninfected with *L. braziliensis *at a 2:1 ratio. After 48 and 96 h, the supernatants were collected for detection of NO **(A) **and superoxide **(B) **using the Griess reaction. The data are expressed as the mean ± SD. Statistical analysis was performed using the Kruskal-Wallis test. (*p *> 0.05).

*L. braziliensis *infection increased NO production and the NO levels tended to be higher at 96 h of infection (Figure 2A). This trend coincided with a significant decrease in the intracellular parasite number in the SC group. There was no statistical difference in NO production among the groups (Figure 2A). Superoxide production by human infected macrophages increased after 96 h of infection but there was no statistical difference in superoxide production between the groups analyzed after 48 and 96 h (Figure 2B).

### Chemokines production by macrophages after infection with *L. braziliensis*

**Figure 3:**
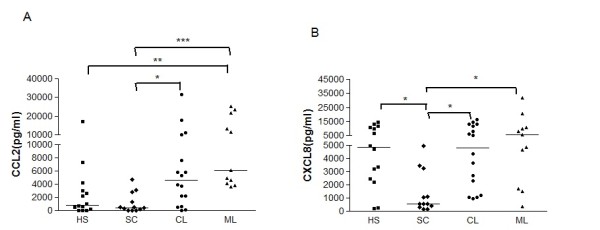
**CCL2 and CXCL8 production by human macrophages infected *in vitro *with *L. braziliensis***. Six-day monocyte derived-macrophages from HS (n = 14), SC (n = 12), CL (n = 16), and ML (n = 11) were infected with *L. braziliensis *at a 2:1 ratio. After 48 h, the supernatants were harvested and the levels of CCL2 (A) and CXCL8 (B) were determined by ELISA. Each symbol represents a different patient, and each line represents the median of a group. For statistical analysis, the Kruskal-Wallis nonparametric test was used. (*p < 0.05, **p < 0.01, *** *p *< 0.001).

**Figure 4:**
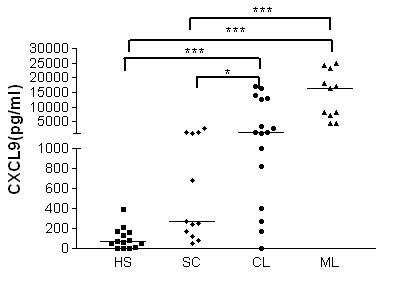
**CXCL9 production by infected human macrophages cultured *in vitro *in the presence of *L. braziliensis***. Six-day monocyte derived-macrophages from HS (n = 14), SC (n = 12), CL (n = 16), and ML (n = 11) were infected *in vitro *with *L. braziliensis *at a 2:1 ratio. After 48 h, the supernatants were harvested and the levels of CXCL9 were determined by ELISA. Each symbol represents a different patient, and each line represents the median of a group. For statistical analysis, the Kruskal-Wallis test was used. (*p < 0.05; ***p < 0.001).

Macrophages from all clinical forms produced low and similar levels of chemokines at 2 h after infection (data not shown). Figures 3 and 4 show the CCL2, CCL3, CXCL8 and CXCL9 levels in the supernatants of *L. braziliensis*-infected macrophages at 48 h of culture. CCL2 production was quite variable. CL macrophages (median 4660 pg/ml, range 0-31618 pg/ml) and ML macrophages (6080 pg/ml, range 3600-25270) produced more CCL2 than SC cultures (median 447 range from 0 to 4700; *p *< 0.05) (Figure 3A). CXCL8 levels in CL (median 4790 pg/ml, 960-16250 pg/ml) and ML (5360 pg/ml, 336-32100 pg/ml) cultures were also higher than in SC individuals (570 pg/ml, 136-4980 pg/ml; *p *< 0.05) (Figure 3B). CCL3 was detected in all groups and did not show significant differences among groups (data not shown). The levels of CXCL9 were significantly higher in CL (1510 pg/ml, 0-17205 pg/ml) and ML patients (16396 pg/ml, 4324-25080 pg/ml) compared with HS (66 pg/ml, 0-390 pg/ml; *p *< 0.001). SC patients produced less CXCL9 (265 pg/ml, 47-2905) than either the ML and CL patients (*p *< 0.001; *p *< 0.05) (Figure 4). At 96 h of culture the levels of chemokines were still high (data not shown) and similar to the levels at 48 h. No differences in LPS-induced CCL2, CXL8 and CXCL9 production were observed between groups (*p *> 0.05) (data not shown).

### Production of TNF-α by macrophages after infection with *L. braziliensis*

**Figure 5:**
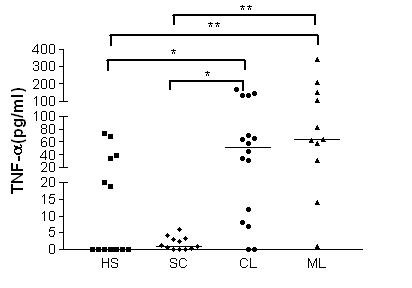
**TNF-α production by human macrophages infected *in vitro *with *L. braziliensis***. Six-day monocyte derived-macrophages from HS (n = 14), SC (n = 12), CL (n = 16), and ML (n = 11) were cultured in the presence of *L. braziliensis *at a 2:1 ratio. After 48 h, the supernatants were harvested and the levels of TNF-α were determined by ELISA. Each symbol represents a different patient, and each line represents the median of a group. For statistical analysis, the Kruskal-Wallis nonparametric test was used. (*p < 0.05; **p < 0.01).

Macrophages from CL, ML, SC and HS patients produced low and similar levels of TNF-α at 2 h after infection (data not shown). At 48 h secreted TNF-α in disease-specific patterns (Figure 5). *Leishmania*-infected macrophages from ML and CL patients secreted more TNF-α (64 pg/ml-ranging 1-341 pg/ml and 52 pg/ml, ranging 0-170 pg/ml, respectively) than macrophages from HS (0 pg/ml, ranging 0-74 pg/ml, *p *< 0.01 and *p *< 0.05). SC macrophages secreted less TNF-α (1.05pg/ml and 0-6.2pg/ml respectively) than cells from ML and CL patients *p *< 0.01 and *p *< 0.05 (Figure 5). At 96 h of culture the levels of TNF-α still were still high (data not shown) and similar to levels at 48 h. No difference (*p *> 0.05) in LPS-induced TNF-α production was observed between groups (data not shown).

## Discussion

Macrophages are important for *Leishmania *infection because they can host the parasite for a long period of time, act as antigen presenting cells and secrete molecules that induce inflammatory response and may kill *Leishmania*. In this study we compared the ability of *L. braziliensis *to infect and survive in macrophages from individuals with different clinical forms of *L. braziliensis *infection. Furthermore, we measured molecules secreted by these cells. Our data clearly show that individuals with SC *L. braziliensis *infection can control parasite growth in the absence of T cells or T cell products through the ability of their macrophages to kill *Leishmania*. Alternatively, macrophages from CL and ML patients allow *Leishmania *survival and produce high amounts of pro-inflammatory chemokines that attract neutrophils, monocytes and activated T cells that induce an inflammatory reaction and contribute to the pathology associated with the *L. braziliensis *infection.

Analyzing macrophage function in individuals with different clinical forms of *L. braziliensis *fills a gap in our understanding of the pathogenesis of leishmaniasis. The Th1-type immune response controls parasite multiplication and dissemination in CL and ML, but it does not eradicate *Leishmania *infection [22]. Moreover, this exaggerated and non-modulated type-1 immune response observed in CL and ML is associated with the production of pro-inflammatory cytokines and tissue damage [7]. In contrast, individuals with SC *L. braziliensis *infection control parasite growth and do not develop the disease, despite producing only low levels of IFN-γ, the main cytokine that activates macrophages to kill *Leishmania*. Therefore, it is likely that in SC individuals the innate immune response plays an important role in the control of parasite growth. Macrophages from sub-clinically infected individuals have been shown to be susceptible to infection by *L. panamensis *than macrophages from patients with recurrent leishmaniasis [23]. In the present study, all groups of macrophages infected with *L. braziliensis *had equal numbers of amastigotes at two hours after infection. We conclude that penetration of *Leishmania *into macrophages is independent of the clinical group from which the macrophage samples were obtained. However, while the percentage of infected cells and number of amastigotes decreased in macrophages from individuals with SC infection, the parasite load in CL and ML remained high, indicating that cells from SC patients have a greater ability to kill parasites than cells from CL and ML patients. The mechanisms used by human macrophages to kill *Leishmania *are not yet well established. NO plays an important role in controlling established murine leishmaniasis infection [24]. A recent study showed that NO production remained unchanged in a co-culture of human neutrophils with macrophages infected with *L. braziliensis *[25]. Our results show that macrophages infected with *L. braziliensis *produced NO and O_2_^-^, although no differences were observed among the different groups, suggesting that macrophage-derived oxidants are not critical in controlling the macrophage infection of *Leishmaniasis *patients by *L. braziliensis*. Analyzing different isolates of *Leishmania *that originated from patients with CL from an endemic area of *L. braziliensis *transmission identified some isolates that were resistant to death by NO, which positively correlated with lesion size [26]. These data suggest that NO production is not the main mechanism used by macrophages to kill *Leishmania*. The interaction between NO and O_2_^- ^leads to the formation of peroxynitrite (ONOO-ROS), which has been shown to have a greater toxic effect *in vitro *in amastigotes when compared to NO [27,28]. Further studies are warranted to evaluate the role of peroxynitrite in the killing of *L. braziliensis*.

Chemokines participate in the inflammatory reaction during *Leishmania *infection. While CCL3 was produced in similar amounts by macrophages from all four groups, the production of CCL2, CXCL8 and CXCL9 after *L. braziliensis *infection was significantly higher in macrophages from CL and ML patients than in those from SC patients. CXCL8 attracts neutrophils to the inflammatory site where they are phagocytized by macrophages. Depending on whether the neutrophils are apoptotic or necrotic, macrophages can control or allow parasite load growth [25,29]. Exogenous addition of CCL2 is a highly specific chemotactic factor for macrophages and T-lymphocytes that enhances *L. infantum *killing by human macrophages [12]. This chemokine showed increased expression in CL lesions compared with control biopsy samples [30]. The expression of CCL2 and CXCL9 was higher in CL tissue cells than in diffuse cutaneous leishmaniasis tissue [11]. CXCL9, previously called a monokine, is induced by IFN-γ (MIG) and has the ability to attract T cells to the site of inflammation. These observations suggest that different chemokines may influence the parasite multiplication, the inflammation and the clinical manifestations of leishmaniasis [12,30]. However, CL and ML macrophages secreted higher levels of CCL2 and CXCL9 but did not kill *Leishmania*, making it more likely that these chemokines are involved more in the inflammatory reaction and tissue damage in CL and ML patients than in protecting them against *L. braziliensis *infection. CXCL9 is secreted in high levels in chronic inflammatory and auto-immune diseases, such as human T cell lymphotropic virus infection and multiple sclerosis. In both cases, CXCL9 is associated with inflammation and the disease pathology [31,32].

TNF-α plays an important role in protecting humans and mice against leishmaniasis [33,34]. However, TNF-α can also mediate the ATL pathology [35-39] Here, we show that macrophages from CL and ML patients produce TNF-α after exposure to *L. braziliensis *infection. Macrophages from CL and ML kill fewer parasites than macrophages from SC, which implies that these cells contribute to the pathology of CL and ML by producing pro-inflammatory cytokines.

The explanation for why CL and ML groups produce higher levels of chemokines and TNF-α is now under evaluation. For instance genetic factors such as polymorphisms in TLR receptors may partly explain this difference. Alternatively, parasite factors may also stimulate the production of chemokines and TNF-α. Recent studies have shown a RNA virus that actives TLR-3 in some *Leishmania *species associated with mucosal disease [40]. This study builds on previous observations that individuals with SC infection can control the *L. braziliensis *parasite despite a poor type-1 immune response [10], while the exaggerated inflammatory response and T cell activation observed in CL and ML contribute to the development of ulcers [35,41]. In this research, we show that macrophages play a pivotal role in the pathogenesis of tegumentary leishmaniasis by decreasing the parasite load in subjects with subclinical *L. braziliensis *infection, even in the absence of T cells and inducing pathology in CL and ML patients, by increasing pro-inflammatory chemokines and cytokines.

## Conclusions

This study shows that macrophages may play a pivotal role in the pathogenesis of human leishmaniasis and that their function may determine the clinical outcome of *L. braziliensis *infection. In SC disease, macrophages efficiently kill parasites and control parasite growth without tissue damage, while macrophages from CL and ML patients allow parasite survival and secrete inflammatory molecules that activate and recruit other cells, leading to pathology. The mechanisms for these findings are not completely understood but provide a fascinating subject for investigation.

## Competing interests

The authors declare that they have no competing interests.

## Authors' contributions

AG, CV, EMC and OB participated equally in the study design. AG, CV, LPC and TL participated in the human macrophages infection and NO detection experiments. AG, CV and CB participated in detecting the levels of chemokines and TNF-α. EMC and OB are the principal investigators of this work and followed the work from the beginning to the end and also participated in the writing of the manuscript. All authors read and approved the final manuscript.

## Pre-publication history

The pre-publication history for this paper can be accessed here:

http://www.biomedcentral.com/1471-2334/12/75/prepub
